# Journey to Transplant: Developing a social support network counselling intervention to improve kidney transplantation

**DOI:** 10.1111/hex.13412

**Published:** 2021-12-23

**Authors:** Hannah D'Cunha, Melissa Partin, Sophie Kurschner, Sauman Chu, Marilyn Bruin, Warren McKinney, Allyson Hart

**Affiliations:** ^1^ Chronic Disease Research Group Hennepin Healthcare Research Institute Minneapolis Minnesota USA; ^2^ Des Moines University College of Osteopathic Medicine Des Moines Iowa USA; ^3^ Department of Medicine, Medical School University of Minnesota (UMN) Minneapolis Minnesota USA; ^4^ College of Design University of Minnesota (UMN) Minneapolis Minnesota USA; ^5^ Division of Nephrology, Department of Medicine Hennepin Healthcare Minneapolis Minnesota USA

**Keywords:** counselling intervention, dialysis, end stage kidney disease, Intervention Mapping, kidney transplant, living kidney donation, social support

## Abstract

**Context:**

Kidney transplant is superior to dialysis for the treatment of end‐stage kidney disease, but accessing transplant requires high patient engagement to overcome barriers. We sought to develop an educational counselling intervention for patients along with their social support networks to help patients access the waiting list.

**Methods:**

Utilizing an Intervention Mapping approach, we established a conceptual framework to develop a behavioural intervention that can be reproduced across kidney transplant centres. The approach includes needs assessment, identifying behavioural determinants and process objectives and integrating targeted behavioural change theory.

**Results:**

The Intervention Mapping process resulted in the development of a group counselling session, titled Journey to Transplant (JtT). This intervention was designed for kidney transplant candidates along with members of their social support networks and guided by a transplant healthcare professional. The session begins with standardized educational information to improve knowledge and normalize emotional barriers to transplant. This education is followed by a tailored counselling intervention, including the presentation of the individual patient's calculated likely outcomes on the kidney transplant waiting list. Finally, JtT incorporates patient and support network goal setting to address the specific barriers for that patient in accessing kidney transplantation.

**Conclusion:**

A systematic Intervention Mapping approach to develop the JtT intervention helps ensure the intervention is efficacious, acceptable and feasible for transplant centres to implement. JtT engages the patient's social support network, targeting known barriers to transplant and utilizing established behaviour change theory to motivate concrete actions to improve the likelihood of kidney transplantation.

**Patient or Public Contribution:**

This study includes a patient and family advisory committee comprised of kidney transplant candidates and their family members to guide the final language and content of the intervention guide, and the conduct of the implementation and pilot testing of the intervention. However, patients and family members were not involved in the intervention mapping development process itself described in this manuscript, which was informed by focus group data from patient and family study participants.

## 1 INTRODUCTION

Kidney transplant is superior to dialysis for the treatment of end‐stage kidney disease^1^, ^2^, ^3^; however, many barriers to transplant exist, which limit access. Accessing transplants requires high patient engagement, as the process requires navigating a challenging system of medical appointments and studies, adhering to burdensome treatments and making complex, high‐stakes decisions. Patient knowledge of options and outcomes on the kidney transplant waiting list is inadequate, which contributes to uninformed decision‐making. Additionally, emotional barriers contribute to limited access to transplantation. Concern about feeling like a burden result in patients' reluctance to ask for living kidney donors or help navigating the system. In addition, fear about the idea of accepting a lower quality deceased donor kidney limits acceptance even when clinical benefit likely exists.^4^, ^5^

A systematic review of patients with chronic kidney disease found that difficulty confronting mortality, lack of information about choices and the timing of information influenced their ability to make informed treatment decisions.^6^ Studies have shown that nephrologists do not consistently discuss mortality risks with patients.^7^, ^8^ Patient‐centred tools have been developed to help patients understand and make decisions regarding transplant options.^4^ However, completion of the pretransplant evaluation process to get onto the waiting list, and utilisation of options, such as living donor transplant, remains suboptimal, resulting in patients dying on dialysis who would have benefitted from transplantation. The current education process inadvertently favours patients with high health literacy, engagement with the healthcare system and resources to navigate the transplant system while concurrently managing their end‐stage kidney disease. To fill this gap, interventions are needed to improve all patients' ability to access kidney transplants.

Intensive home‐based group counselling with a behavioural psychologist has been shown to improve rates of living kidney donation.^9^ However, this approach is not feasible for most transplant programmes. Additional promising coaching and education interventions have been developed, though they target the individual patient and often require multiple follow‐up visits, potentially complicating implementation.^10^ A review on strategies to increase living kidney donation concluded that targeting transplant candidates and their social support networks is a promising point of intervention,^11^ such as that employed by the TALKS intervention.^12^ However, this social worker‐directed intervention was focused on living donation, and while social workers are often trained in counselling, they may not be able to provide the additional medical counselling that patients and their social support networks need to navigate the Journey to Transplant. The Kidney COACH program^13^ also seeks to activate a member of the social support network to seek living donation, but is targeted to one support member of a waitlisted patient, does not seek to improve overall access to transplant and is more standardized information than individualized to the barriers faced by a specific patient. We sought to develop a tailored group counselling intervention to improve access to kidney transplants that targets patients and their social support members, would be feasible to implement in transplant clinics and relied on evidence‐based theories of behaviour change.

## 2 METHODS

We used an Intervention Mapping approach to incorporate data on facilitators and barriers to accessing kidney transplants into an intervention with adult kidney transplant candidates and their social support networks. This approach was comprised of six steps, each of which integrated theory and evidence into an intervention.^14^ The protocol served as a blueprint for designing, implementing and evaluating a theory‐based intervention.

### 2.1 Step 1: Logic model of the problem

We created a needs assessment and a logic model of the problem.^14^ To conduct our needs analysis, we both reviewed existing literature and prospectively collected qualitative data from focus groups of patients seeking transplants as well as their friends and family members. These data were used to identify specific barriers and unmet needs for kidney transplant candidates and their social support networks, and how these barriers contribute to decreased access to kidney transplantation. Qualitative data were collected from 13 focus groups: 6 focus groups with a total of 48 kidney transplant candidates and 7 focus groups with 38 friends and family members, conducted in Minnesota and Georgia. Methods and analyses for these groups have been previously described.^5^, ^15^, ^16^ Initial patient focus groups were designed to understand what patients understood about getting a kidney transplant and likely outcomes on the waiting list. Later focus groups transitioned to social support networks as well as additional patient focus groups based on the theme of the value of having an engaged social network that emerged from early groups. These later focus groups explored the barriers and facilitators to engaging friends and family as well as what information and support would be helpful. Themes from these groups informed the Intervention Mapping process outlined below, including the development of process objectives, to design an evidence‐based social support intervention to improve access to kidney transplants.

### 2.2 Step 2: Programme outcomes and objectives

Key determinants and process objectives to achieve access to transplants were identified on both the individual and environmental levels. After identifying key determinants and process objectives, we created matrices to connect these determinants and processes to identify change objectives. The change objectives serve as actionable goals, which the intervention must aim to target. We then created a logic model of change. The logic model of change illustrates how the change objectives target determinants to address the process objectives, subsequently affecting health behaviours and outcomes.

### 2.3 Step 3: Programme design

We identified social‐behavioural theories to inform evidence‐based change methods targeting the change objectives identified in Step 2. Behavioural change methods were selected based on identified theories and practical applications were developed to deliver these change methods.

### 2.4 Step 4: Programme production

We integrated the behavioural change methods into an organized programme.^14^ During this phase, we developed prototypes, outlines and scripts for programme materials and sessions. These scripts were reviewed with a 12‐member patient and family advisory panel for additional feedback.

### 2.5 Step 5: Programme implementation plan

An implementation plan requires plans for the adoption, implementation and sustainability of the intervention. During this step, we identified the logistics of implementation, including who will implement the intervention, at what time point in the evaluation process and how the programme will be implemented at the transplant clinic, along with associated feasibility pilot planning. Implementation challenges were also reviewed with the patient and family advisory panel.

### 2.6 Step 6: Evaluation plan

An evaluation plan was then designed to conduct outcome evaluations of the intervention through the development of a survey measuring the specific determinants targeted by the intervention. This evaluation aims to assess whether or not the change objectives of the programme were met. Process evaluation plans were developed to ensure effective programme implementation.

## 3 RESULTS

### 3.1 Logic model of the problem

Based on previous literature and our qualitative data suggesting that modifiable determinants exist for both patients and their friends and families, we chose to focus on both the interpersonal level, as well as the environmental level, specifically the social support groups of kidney transplant patients. Specific needs and barriers of patients and families have been previously published^5^, ^16^, ^17^ but broad themes from these analyses include (1) patients with advanced kidney disease are overwhelmed by the information they must process and by the burden of dialysis treatments, (2) patients want their social support networks to understand what they are going through, but do not want to have to relay all medical information, (3) patients feel like a burden and therefore make decisions they feel will spare their friends and family from having to do more related to their disease, (4) families want to be more involved but don not always know what to do or how to help and (5) feeling uninformed about the patient's disease and treatment options cause more anxiety in friends and family members.

### 3.2 Programme outcomes and objectives

We identified eight high‐level process objectives involved in achieving a kidney transplant for the individual (patient) level: (1) ask a living donor (if available), (2) know and understand the process of getting a kidney transplant, (3) tell living donor how to navigate donation process, (4) follow through on medical appointments and recommendations, (5) engage social support network to help with the transplant evaluation process, (6) accept higher risk deceased donor organs when medically appropriate (if no living donor available), (7) communicate proactively with the transplant team to keep the process moving forward and (8) develop an effective relationship with transplant providers. These were further broken down into more specific change objectives that could be targeted through behavioural intervention approaches. The environmental/intrapersonal level process objectives identified for social support network members included (1) engaging with the patient to help with the transplant process, (2) feeling comfortable giving help, (3) understanding the transplant process and (4) being an active part of the care team. Determinants on the individual level to achieve these process objectives include knowledge (including risk awareness and perception), self‐efficacy, attitudes and beliefs and social support. Determinants identified on the environmental/interpersonal level included knowledge, self‐efficacy, motivation/intention and social influence.

**Figure 1:**
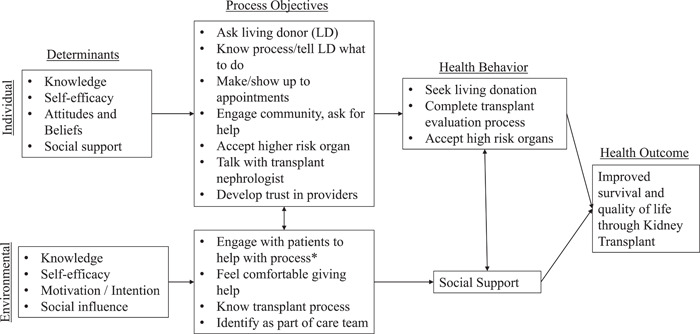
Logic model illustrating the determinants and process objectives identified to influence health behaviours and influence the outcome of improved survival and quality of life for patients with end‐stage kidney disease

Determinants and process objectives were placed into matrices to identify a change objective for each determinant‐objective intersection (Supporting Information Material 1). After identifying determinants and process objectives, a logical model of change was created to illustrate how the determinants and objectives aim to influence the environmental conditions (through the social support networks) and individual patient health behaviour and outcomes (Figure 1). After creating the logic model of change, we identified theories of behaviour change to target each change objective. A behaviour change theory was identified for each behaviour change objective and potential methods of change were then identified for each objective. Tables mapping the process objective to behavioural determinants, behaviour change theories and methods were developed for both the individual (patient) and environmental (social support network members) levels. An overview of the mapping tables for the patient and environmental levels is shown in Table 1.

**Table 1:** Summary of overarching explanatory and behaviour change theories mapped onto the process and change objectives at the patient and environmental (social support network) levels

Process objectives	Change objectives	Behavioural determinants	Behaviour Explanatory Theory	Behaviour Change Theory	Constructs	Potential methods
Patient level						
Ask living donor	Describe the benefits and risks of living donation	Knowledge	SCT	SCT, DSF	Knowledge	Using imagery Discussion Elaboration Decision aid
	Feel confident in asking the living donor for organ	Self‐efficacy	SCT	SCT	Self‐efficacy	Self re‐evaluation
	Feel sure about asking the living donor to donate the organ	Decisional conflict	SCT	DSF	Attitudes and expectations Values Uncertainty	Consciousness raising Personalized risk Framing
Understand the transplant process	Describe the process of getting listed and transplanted	Knowledge	SCT	SCT	Knowledge	Using imagery Discussion
	Understand benefits, risks and outcomes of different options for transplant (living vs. deceased, increased infectious risk donor organs, acceptance of lower quality deceased donor organs	Decisional conflict	HBM	DSF	Knowledge Attitudes and expectations Support Resources	Discussion Using imagery Elaboration Fear arousal
Tell living donor what to do	Describe the steps living donors need to take to donate	Knowledge	SCT	SCT	Knowledge	Discussion Elaboration
	Feel confident in communicating what you need the living donor to do	Self‐efficacy	SCT	SCT	Self‐efficacy	Self re‐evaluation Improving physical and emotional states
Make and attend follow‐up appointments	Feel confident that you can show up to appointments and follow‐ups	Self‐efficacy	SCT	SCT	Self‐efficacy	Self‐monitoring of behaviour Provide contingent rewards Goal setting
Engage the community to help with the process	Feel confident engaging with your community in the transplant process	Self‐efficacy	SCT	SCT	Self‐efficacy	Providing opportunities for social comparison Verbal persuasion Public commitment
	Feel like you have enough support and advice from the community to make an informed decision	Decisional conflict	Social support	DSF, social support	Perceived support	Mobilizing social support Providing opportunities for social comparison Peer education
	Engage family and friends in transplant process: invite to appointments, etc.	Social support	Social support	DSF, social support	Perceived support	Public commitment Enhancing network linkages Mobilizing social support
Accept an increased‐risk organ when medically appropriate	Describe the benefits and risks of accepting an increased‐risk organ	Knowledge	SCT HBM	SCT	Knowledge	Chunking Elaboration Decision aid
	Feel sure that accepting a higher risk organ is the best choice for you	Decisional conflict	HBM	DSF	Perceived susceptibility and severity, perceived benefits Attitudes and expectations	Direct experience Information about others' approval Mobilizing social support
Communicate with the transplant team	Feel comfortable and confident proactively communicating with a transplant nephrologist	Self‐efficacy	Social Support	Social Support, SCT	Perceived support Self‐efficacy	Mobilizing social support Enhancing network linkages
	Consider transplant team a part of your care team	Social support	Social support	Social support	Perceived support	Cultural similarity Mobilizing social support Enhancing network linkages Peer education
Develop a trusting relationship with the provider	Feel comfortable with your provider	Self‐efficacy	Social Support	Social Support	Perceived support Received support	Direct experience Cultural similarity Enhancing network linkages
Environmental (social support network) level						
Understand the transplant process	Know the pros and cons of transplant vs. dialysis	Knowledge	HBM	DSF	Attitudes and expectations Values Uncertainty	Fear arousal norming Framing and environmental re‐evaluation
	Know the steps and barriers to getting transplanted	Knowledge	HBM	DSF	Knowledge	Information
	Understand medical/treatment decisions to be made	Decisional conflict	HBM	DSF	Attitudes and expectations Values Uncertainty	Decision aids
Feel comfortable providing help to the patient	Engage with your patient and medical community in the transplant process, be an active part of the care team	Self‐efficacy	SCT	SCT	Self‐efficacy Collective efficacy	Providing opportunities for social comparison Mobilizing social support Verbal persuasion Public commitment
	Feel like you have enough support and advice from medical teams to support informed decision	Decisional conflict	Social support	DSF, social support	Perceived support	Education Mobilizing social support Enhancing network linkages through education
	Identify specific barriers for your patient and steps to overcome them	Knowledge	Social support	Social Norms Theory	Knowledge Attitudes and expectations Self‐efficacy	Snowballing Goal‐setting Mobilizing social network Enhancing network linkages through education
		Social support				
		Self‐efficacy				
	Feel confident navigating difficult discussions with the patient who is a friend or family member	Self‐efficacy	Social Norms Theory	Social Norms Theory	Attitudes and expectations	Normalisation
Seek out living donors	Describe the steps living donors need to take to donate	Knowledge	SCT	SCT	Knowledge	Discussion Elaboration
	Feel confident in communicating what you need the living donor to do	Self‐efficacy	SCT	SCT	Self‐efficacy	Information Discussion Elaboration Improving physical and emotional states
	Ask potential living donors	Self‐efficacy	SCT	SCT, changing social norms	Self‐efficacy Social support	Mobilizing social support Peer–peer interaction Snowballing Goal‐setting Theory
		Social support				

*Note*: This sample of process and change objectives were identified by the research team as the most amenable to a transplant centre intervention.

Abbreviations: DSF, decision support framework; HBM, Health Behavioural Model; SCT, Social Cognitive Theory.

### 3.3 Programme design, production, implementation and evaluation

**Figure 2:**
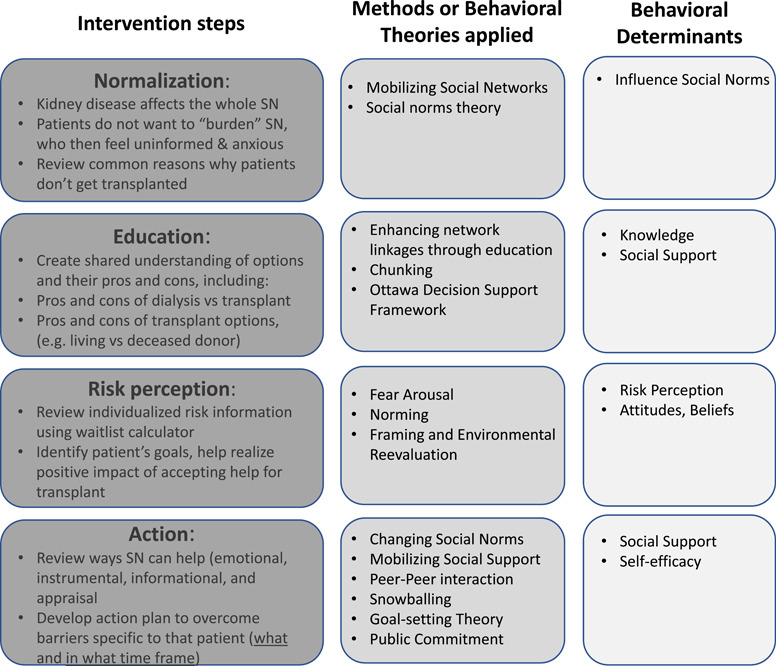
Overview of the intervention steps, their theoretical foundations and the behavioural determinants targeted. SN, social network

Reviewing the potential behavioural change theories and methods, we selected a number of feasible change methods and developed a group counselling and education session titled Journey to Transplant, designed for a kidney transplant candidate with members of their social support networks and guided by a transplant healthcare professional. Using the selected change methods, we developed an outline for the intervention in parallel with plans for programme implementation and programme materials, including a booklet in which participants can take notes (Figure 2, Table S2). The session is broken into a section designed to improve knowledge and perception of risk that is highly standardized, followed by more individualized content. Importantly, the intervention includes a presentation of the patients' calculated likely outcomes on the kidney transplant waiting list to help with the issue of risk perception and expectations. This information is followed by sections focused on patient goal setting and the social support network goal setting to address barriers and needs specific to that of the patient and their friends and family members.

We then identified the logistics of implementation, along with an evaluation plan specifically measuring the behavioural change objectives we targeted (Table S2). To ensure that the intervention is relatively uniform, we developed an intervention guide with a general script, similar to a focus group guide. Implementation concerns for the development of this group education session include patient and social support network distance from the transplant centre, transportation concerns, logistical concerns regarding scheduling around work and childcare, as well as concerns with gathering a group together in person in the era of COVID‐19. Therefore, the intervention was designed to be a virtual group meeting, with the educational workbook mailed out to participants in advance to follow along and take notes. The programme evaluation consists of a pre–post survey design, aimed at measuring the determinants of interest in both the patient and their social support network. Programme implementation barriers will be further evaluated in a pilot feasibility trial of the intervention.

## 4 DISCUSSION

Using an Intervention Mapping process, we developed a conceptual framework for a behavioural intervention to improve access to kidney transplants, and from this a group counselling intervention with patients and their social support network members, the Journey to Transplant intervention. This approach includes a rigorous series of steps to ensure that stakeholder needs are addressed, applying established methods of behaviour change and incorporating plans to measure efficacy and feasibility early in the process. This process has helped to construct a standardized but individualized, theory‐driven intervention that can be reproduced and utilized in the clinical setting.

This intervention is designed to address a significant gap in the care of patients with advanced chronic kidney disease. The superiority of kidney transplants over dialysis for the treatment of end‐stage kidney disease in terms of both mortality and quality of life is well‐established; however, access to kidney transplants remains suboptimal. Although much of this limited access can be explained by a supply–demand mismatch of donor organs, many patient‐ and provider‐level factors can improve a patient's likelihood of kidney transplantation. In addition, significant racial and socioeconomic inequities in access to kidney transplants persist, particularly among African Americans.^16^, ^18^ We sought to design an intervention that would draw on the untapped resource of a patient's social support network, while also levelling the knowledge playing field for patients and social support networks who may not have as much healthcare literacy. To this end, we intentionally recruited African American candidates to ensure that our data on the needs of transplant candidates and their social support networks did not only represent the needs of White patients.

In utilizing the Intervention Mapping approach, we sought to maintain a focus throughout the intervention development process on feasibility, implementation and dissemination. Several well‐designed interventions have been found in clinical studies to improve access to kidney transplants, however, are very resource‐intensive interventions that are difficult for many transplant centres to implement.^9^ The intervention was designed to be guided by a healthcare professional with knowledge of transplant, such as a transplant nurse coordinator, but not necessarily trained in behavioural counselling or theory in order to improve feasibility. The intervention was also informed and reviewed by a patient and family advisory panel to guide language, content and implementation.

The intervention includes an individualized risk calculator to help patients understand their own likely outcomes on the kidney transplant waiting list if they do not have a living donor, given their medical characteristics and region. While this calculator was intended to assist with knowledge and risk perception, usability testing done with patients during the development of this online tool highlighted its potential for fear arousal, given the often‐poor outcomes for patients with multiple other comorbidities and often high likelihood of dying while on the waiting list. While patients reported wanting this information even though it was difficult, it reinforced the need to follow this potentially distressing information with a discussion about the concrete actions and decisions that patients and their social support networks can make to improve the likelihood of kidney transplantation, a necessary component when fear arousal is utilized in a behavioural change intervention. We had the opportunity to complete four usability focus groups with a national sample of kidney transplant recipients, as well 15 usability tests with candidates, to review the ‘pros and cons’ and calculator education content.^19^ Although this was in‐person rather than virtual, the language, flow of information and facilitator check‐ins that are needed were developed from these usability tests. However, the transition to a virtual delivery format will need to be pilot tested.

The decision to develop a social support network intervention also necessarily excludes patients who do not have a social support network or patients who are not interested in engaging or including their network. Although this is certainly a concern, no intervention will be relevant to every patient. This issue was also discussed with our patient and family advisory panel, with their recommendation to help patients expand their definition of their support network beyond traditional family members if possible when recruiting patients for the intervention in future pilot testing. In addition, the proportion and characteristics of patients who report a lack of support network members as a reason for not participating will be an important measure for any trials on the efficacy of the intervention. Similarly, the decision to proceed with a virtual intervention raises concerns about access to devices and the internet. Assistance with access to devices and tracking of how often this prevents participation will be another critical measure in future trials to ensure that this intervention does not worsen rather than improve disparities in access to transplants by socioeconomic status. Fortunately, the pandemic has forced healthcare centres to learn how to provide virtual care, which gives this novel counselling approach a jumpstart on its path to usual clinical care if it proves to be efficacious.

Having developed the intervention materials and guide, the critical next step is feasibility and pilot testing. While our systematic, theory‐based approach increases the likelihood that the intervention will be efficacious, our intervention development is also grounded in an iterative, ‘design thinking’ approach that enables us to make continuous improvements along the way. At this stage, enroling a small number of patients and their social support networks in a pilot trial will further assess the content, pilot the measurement tools of knowledge, attitude and behaviour and provide information on how this intervention should fit into usual clinical care provided by nephrologists and transplant nurse coordinators before finalizing the intervention script and materials that can be tested for efficacy in improving access to transplant in a larger trial.

Our study has important limitations. The focus groups from which we drew heavily for our needs assessment in the intervention mapping process were conducted from two geographical areas in the United States. While these two regions, Minneapolis and Atlanta, were selected to represent a range of experiences and a racially diverse group of participants, the needs of patients from other regions or ethnicities may not be fully represented or addressed by this intervention. Furthermore, we included only English‐speaking populations, therefore additional unaddressed cultural and language barriers may need to be addressed if this intervention is applied to other populations. The ‘logic model of change’ used in intervention mapping is a model focused on behaviour determinants and processes (whether that of the patient or the individuals in their environment, in this case, the social support network). Therefore, socioeconomic status and insurance are not explicitly included in this particular model but do likely impact access to transplants and are therefore addressed later in the problem‐solving phase of the intervention. Finally, usability testing of this intervention or its efficacy in improving rates of transplantation has not been established. The feasibility pilot study of the intervention is currently underway in Minnesota and Georgia, but a multicentre randomized trial will be required to evaluate the intervention's effect on transplant rates.

## 5 CONCLUSION

Emotional and educational barriers often limit access to kidney transplantation. An Intervention Mapping approach can provide a conceptual framework to help design behavioural interventions that keep in mind feasibility, implementation and dissemination. Interventions that aim to improve limitations in kidney transplantation should incorporate the untapped resource of a patient's social support network, work to enhance patient and social support network knowledge about transplant and address concrete actions as well as decisions that patients and their social support networks can make to improve the likelihood of kidney transplantation.

## CONFLICT OF INTERESTS

The authors declare that there are no conflict of interests.

## AUTHOR CONTRIBUTIONS

The authors confirm contribution to the paper as follows: *Study conception and design*: Hannah D'Cunha, Melissa Partin, Sauman Chu, Marilyn Bruin, Allyson Hart; *data collection*: Hannah D'Cunha, Melissa Partin, Sauman Chu, Marilyn Bruin, Allyson Hart; *analysis and interpretation of results*: Hannah D'Cunha, Melissa Partin, Marilyn Bruin, Sophie Kurschner, Warren McKinney, Allyson Hart; *draft manuscript preparation*: Hannah D'Cunha, Melissa Partin, Marilyn Bruin, Sophie Kurschner, Warren McKinney, Allyson Hart. All authors reviewed the results and approved the final version of the manuscript.

## Supporting information

Supporting information.

Supporting information.

## Data Availability

Data described in this manuscript are available from the authors on reasonable request.
