# Supplementary material for: Journey to Transplant: Developing a social support network counselling intervention to improve kidney transplantation
Source: Health Expect. 2021 Dec 23;25(2):648–58. doi: 10.1111/hex.13412 (PMC8957747; doi:10.1111/hex.13412)
Supplement: Supplementary file 1 — Supporting information. [file HEX-25--s001.docx]

| **Supplemental Table 2. Detailed intervention outline with behavior change theory and behavioral determinant targeted** | | | |
| --- | --- | --- | --- |
| **Section Focus** | **Outline** | **Method or Theory of Behavior Change applied** | **Determinant /Construct targeted** |
| **Knowledge** | **I. OVERVIEW**  1. Introductions  2. Background information: What we’ve learned  - Kidney disease is a disease of the entire family  - Our previous research found:   - Families want to be more involved but don’t always know what to do or how to help, feeling uninformed causes anxiety - People with kidney disease don’t want to have to relay all medical information or ask their friends and families to do more, feel like a burden   - Hard, complicated process: common reasons why patients don’t get transplanted:   - Don’t complete the medical / evaluation process - Get too sick for transplant while waiting for a deceased donor kidney - Difficulty following medical recommendations and making it to appointments   - Potentially hard conversations about risks and outcomes, feel free to step out and take breaks as needed  **Session goal: We will discuss barriers to transplant and ways patients and families can try to overcome them** | Mobilizing Social Networks / Social norms theory  [Discussing common perceptions and barriers that other patients and families reported makes these feel normal and therefore confrontable] | Influence social norms |
|  | **II. KNOWLEDGE OF OPTIONS**   - Review the information pages of the shared decision aid with pros and cons | - Advanced organizers for info processing - Enhancing network linkages (through training / education) - Discussion | - Knowledge - Social support - knowledge |
|  | **III. Review calculator of your likely outcomes on the deceased donor waiting list**   - Allow time to process, break if needed | - Fear arousal - Norming | - Knowledge: Risk awareness and perception - Attitudes / beliefs / outcome expectations |
| **Patient Goal-setting** | **IV. DISCUSSION OF PATIENT GOALS:**  **What is important to you?**  **What are you hoping to get from your treatment for kidney disease?**  e.g. watch grandkids grow up, go back to work, travel, live as long as possible etc.  **What kind of help do you think you need?** | - Framing and environmental reevaluation [realize positive impact on self and family of accepting help, getting transplanted | - Attitudes / beliefs / outcome expectations |
| **Social Support Goal-setting** | **V. Review Social Support methods:**  - Emotional  - Instrumental / tangible [rides, childcare]  - Informational [advice, information processing]  - Appraisal [advice, feedback on choices] | - Enhance network linkages  - change social norms  - mobilizing social support  - peer-peer interaction snowballing | - Social Support  - Self-efficacy |
|  | **VI. NEXT STEPS**  1. Encouragement: This is hard but doable!  2. Goal setting –behavior focused (What will I try to do?):   - “Plan to think more about / discuss what I can do” is a valid response - Within what time frame? - Each person writes down and read aloud*   3. Plan coping responses – have them list barriers to getting transplanted and how they can collectively try to overcome those barriers  4. Send summary of goals to transplant coordinator | - Verbal persuasion - Goal-setting theory - Public commitment | - Self-efficacy |
