# Supplementary material for: Journey to Transplant: Developing a social support network counselling intervention to improve kidney transplantation
Source: Health Expect. 2021 Dec 23;25(2):648–58. doi: 10.1111/hex.13412 (PMC8957747; doi:10.1111/hex.13412)
Supplement: Supplementary file 2 — Supporting information. [file HEX-25--s002.docx]

**Supplemental Material 1.** Patient and family survey evaluation measuring behavioral change objectives.

**Patient Survey**

1. A patient on dialysis has the same level of kidney functioning as a patient with a transplanted kidney.

True

False

Don’t know

1. In general, patients can live at least 5 years longer with a kidney transplant than if they stayed on dialysis.

True

False

Don’t know

1. In general, most people on dialysis are happier with the quality of their lives than people with transplants.

True

False

Don’t know

1. Patient have better health outcomes if they receive a transplant before starting dialysis.

True

False

Don’t know

1. If a patient waits long enough on the waitlist, a matching kidney from someone who has died will definitely become available.

True

False

Don’t know

1. About what percentage of all transplanted kidneys function for at least one year?

50%

75%

90%

Don’t know

1. Nationally, how long do patients generally wait on the wait list for a kidney from someone who has died?

<1 year

1-2 years

3-5 years

>5 years

Don’t know

1. Compared to transplants from donors who have died, how long do transplants from living donors last?

Shorter

Longer

Same

Don’t know

1. What is the chance that a living donor or recipient would dies undergoing surgery?

<1%

3%

10%

25%

Don’t know

1. Do you feel SURE about the best choice for you?

Yes

No

1. Do you know the benefits and risks for getting a kidney transplant?

Yes

No

1. Are you clear about which benefits and risks matter most to you?

Yes

No

1. Do you have enough support and advice to make a choice?

Yes

No

1. I have a positive outlook toward life.

Strongly agree

Agree

Disagree

Strongly disagree

1. I have short and/or long-range goals.

Strongly agree

Agree

Disagree

Strongly disagree

1. I feel all alone.

Strongly agree

Agree

Disagree

Strongly disagree

1. I can see possibilities in the midst of difficulties.

Strongly agree

Agree

Disagree

Strongly disagree

1. I have faith that gives me comfort.

Strongly agree

Agree

Disagree

Strongly disagree

1. I feel scared about my future.

Strongly agree

Agree

Disagree

Strongly disagree

1. I can recall happy/joyful times.

Strongly agree

Agree

Disagree

Strongly disagree

1. I have a deep inner strength.

Strongly agree

Agree

Disagree

Strongly disagree

1. I am able to give and receive caring/love.

Strongly agree

Agree

Disagree

Strongly disagree

1. I have a sense of direction.

Strongly agree

Agree

Disagree

Strongly disagree

1. I believe that each day has potential.

Strongly agree

Agree

Disagree

Strongly disagree

1. I feel my life has value and worth.

Strongly agree

Agree

Disagree

Strongly disagree

1. How confident are you filling out medical forms by yourself?

Extremely

Quite a bit

Somewhat

A little bit

Not at all

**Family Survey**

1. A patient on dialysis has the same level of kidney functioning as a patient with a transplanted kidney.

True

False

Don’t know

1. In general, patients can live at least 5 years longer with a kidney transplant than if they stayed on dialysis.

True

False

Don’t know

1. In general, most people on dialysis are happier with the quality of their lives than people with transplants.

True

False

Don’t know

1. Patient have better health outcomes if they receive a transplant before starting dialysis.

True

False

Don’t know

1. If a patient waits long enough on the waitlist, a matching kidney from someone who has died will definitely become available.

True

False

Don’t know

1. About what percentage of all transplanted kidneys function for at least one year?

50%

75%

90%

Don’t know

1. Nationally, how long do patients generally wait on the wait list for a kidney from someone who has died?

<1 year

1-2 years

3-5 years

>5 years

Don’t know

1. Compared to transplants from donors who have died, how long do transplants from living donors last?

Shorter

Longer

Same

Don’t know

1. What is the chance that a living donor or recipient would dies undergoing surgery?

<1%

3%

10%

25%

Don’t know

1. I am confident that I could deal efficiently with unexpected events.

Never

Almost never

Sometimes

Fairly often

Very often

1. I intend to help my friend or family member get through the transplant process.

Strongly agree

Agree

Neither agree nor disagree

Disagree

Strongly disagree

1. Are you seriously thinking about helping my friend or family member get through the transplant process?

Yes

No

1. How confident are you filling out medical forms by yourself?

Extremely

Quite a bit

Somewhat

A little bit

Not at all
